# Soil nutrient maps of Sub-Saharan Africa: assessment of soil nutrient content at 250 m spatial resolution using machine learning

**DOI:** 10.1007/s10705-017-9870-x

**Published:** 2017-08-02

**Authors:** Tomislav Hengl, Johan G. B. Leenaars, Keith D. Shepherd, Markus G. Walsh, Gerard B. M. Heuvelink, Tekalign Mamo, Helina Tilahun, Ezra Berkhout, Matthew Cooper, Eric Fegraus, Ichsani Wheeler, Nketia A. Kwabena

**Affiliations:** 1 e-mail: johan.leenaars@wur.nl; 2 e-mail: gerard.heuvelink@wur.nl; 3 World Agroforestry Centre (ICRAF), Nairobi, Kenya e-mail: K.SHEPHERD@cgiar.org; 4 The Earth Institute, Columbia University, New York, NY, USA e-mail: markusgwalsh@gmail.com; 5 Selian Agricultural Research Inst, Arusha, Tanzania; 6 Ethiopian Agricultural Transformation Agency (ATA), Addis Ababa, Ethiopia e-mail: Tekalign.Mamo@ata.gov.et; 7 e-mail: Helina.Tilahun@ata.gov.et; 8 PBL Netherlands Environmental Assessment Agency, The Hague, The Netherlands e-mail: Ezra.Berkhout@pbl.nl; 9 Conservation International, Arlington, VA, USA e-mail: mcooper@conservation.org; 10 e-mail: efegraus@conservation.org; 11 Envirometrix Inc, Wageningen, The Netherlands e-mail: ichsani@envirometrix.net; 12 CSIR-Soil Research Institute, PMB Kwadaso, Kumasi, Ghana e-mail: ka.nketia@csir-soilresearch.org

**Keywords:** Macro-nutrients, Micro-nutrients, Random forest, Machine learning, Soil nutrient map, Spatial prediction, Africa

## Abstract

Spatial predictions of soil macro and micro-nutrient content across Sub-Saharan Africa at 250 m spatial resolution and for 0–30 cm depth interval are presented. Predictions were produced for 15 target nutrients: organic carbon (C) and total (organic) nitrogen (N), total phosphorus (P), and extractable—phosphorus (P), potassium (K), calcium (Ca), magnesium (Mg), sulfur (S), sodium (Na), iron (Fe), manganese (Mn), zinc (Zn), copper (Cu), aluminum (Al) and boron (B). Model training was performed using soil samples from ca. 59,000 locations (a compilation of soil samples from the AfSIS, EthioSIS, One Acre Fund, VitalSigns and legacy soil data) and an extensive stack of remote sensing covariates in addition to landform, lithologic and land cover maps. An ensemble model was then created for each nutrient from two machine learning algorithms— random forest and gradient boosting, as implemented in R packages ranger and xgboost—and then used to generate predictions in a fully-optimized computing system. Cross-validation revealed that apart from S, P and B, significant models can be produced for most targeted nutrients (R-square between 40–85%). Further comparison with OFRA field trial database shows that soil nutrients are indeed critical for agricultural development, with Mn, Zn, Al, B and Na, appearing as the most important nutrients for predicting crop yield. A limiting factor for mapping nutrients using the existing point data in Africa appears to be (1) the high spatial clustering of sampling locations, and (2) missing more detailed parent material/geological maps. Logical steps towards improving prediction accuracies include: further collection of input (training) point samples, further harmonization of measurement methods, addition of more detailed covariates specific to Africa, and implementation of a full spatiotemporal statistical modeling framework.

## Introduction

Sub-Saharan Africa (SSA) has over 50% of the world’s potential land for cultivation, yet only a small portion of this land satisfies conditions for agricultural production from cropping (Lal 1987; Jayne et al. 2010). Although the proportion of arable land in SSA has been steadily growing since 1950’s, currently only 9% of SSA is arable land and only 1% is permanently cultivated^1^. Current cropping yields in Sub-Saharan Africa are low, often falling well short of water-limited yield potentials (Jayne et al. 2010). This underperformance is due to number of factors: soil nutrient deficiencies, soil physical constraints, pests and diseases and sub-optimal management. Whilst it is well established that nutrient deficiencies are constraining yields in SSA (Giller et al. 2009),only limited information is available on soil nutrient contents and nutrient availability. Only very general (approximate) maps of soil micro-nutrients are at the moment available for the whole continent (see e.g. Kang and Osiname 1985; Roy et al. 2006 and/or Alloway 2008).

The Africa Soil Information Services project has recently developed a gridded Soil Information System of Africa at 250 m resolution showing the spatial distribution of primary soil properties of relatively stable nature, such as depth to bedrock, soil particle size fractions (texture), pH, contents of coarse fragments, organic carbon and exchangeable cations such as Ca, Mg, Na, K and Al and the associated cation exchange capacity (Hengl et al. 2015, 2017). These maps were derived from a compilation of soil profile data collected from current and previous soil surveys. There is now a growing interest in applying similar spatial prediction methods to produce detailed maps of soil nutrients (including micro-nutrients) for SSA, in order to support agricultural development, intensification and monitoring of the soil resource (Kamau and Shepherd 2012; Shepherd et al. 2015; Wild 2016). Detailed maps of soil nutrients, including micronutrients, are now possible due to the increasing inflow of soil samples collected at field point locations by various government and/or NGO funded projects: e.g. by projects supported by the National Governments of Ethiopia, Tanzania, Kenya, Uganda, Nigeria, Ghana, Rwanda, Burundi and others; and by organizations such as the Bill and Melinda Gates Foundation (Leenaars 2012; Shepherd et al. 2015; Towett et al. 2015; Vågen et al. 2016) and similar, as well as by the private sector.

We present here results of assessment of nutrient content for a selection of soil nutrients for SubSaharan Africa at a relatively detailed spatial resolution (250 m). Our overarching objective was to map general spatial patterns of soil nutrient distribution in Sub-Saharan Africa. This spatial distribution could then potentially be used as:

- inputs for pan-continental soil-crop models,
- inputs for large scale spatial planning projects,
- inputs for regional agricultural decision support systems,
- general estimates of total nutrient content against which future human-induced or natural changes may be recognized and measured, and as
- prior information to guide more detailed soil sampling surveys.

As the spatial prediction framework we use an ensemble of random forest (Wright and Ziegler 2016) and gradient boosting (Chen and Guestrin 2016) machine-learning techniques, i.e. a weighted average formula described in Sollich and Krogh (1996). As inputs to model building we use the most complete compilation of soil samples obtainable and a diversity of soil covariates (primarily based on remote sensing data).

We generate predictions of individual nutrients, then look at the possibilities of delineating nutrient management zones using automated cluster analysis. At the end, we analyze whether the produced predictions of soil nutrients (maps) are correlated with field measured crop yields based on field trials.

## Materials and methods

### Soil nutrient samples

As input data, we used a compilation of georeferenced soil samples (ca 59,000 unique locations) processed and analyzed consistently using the Mehlich 3 method and/or equivalent (Eckert and Watson 1996; Roy et al. 2006). Data sets used for model building include:

- AfSIS (Africa Soil Information Service) Sentinel Sites: 18,000 soil samples at 9600 locations i.e. 60 sites of 10 by 10 km (Walsh and Vågen 2006; Vågen et al. 2010). Samples were taken in the period 2008–2016 at 0–20 and 20–50 cm soil depth intervals; analyzed by mid-infrared (MIR) diffuse reflectance spectroscopy based on calibration points from 960 samples (10%) analyzed by conventional wet chemistry including Mehlich-3, and thermal oxidation for org. C and total N. Sentinel Sites were designed to cover all of the agro-ecological regions in SSA and therefore should provide a good range of covariates at each location.
- EthioSIS (Ethiopia Soil Information Service): 15,000 topsoil samples (0–20 cm) from Ethiopia analyzed by conventional wet chemistry including Mehlich-3. The majority of samples was collected in the period 2012–2015.
- The Africa Soil Profiles database compiled for AfSIS: over 60,000 samples of 18,500 soil profiles collected from on average four depth intervals to on average 125 cm depth in period 1960–2010 (mainly 1980–1990) and 40 countries, with C, N, K, Ca and Mg available for nearly all points, P for one third of the points and micro-nutrients for ca 20% of points (Leenaars 2012).
- International Fertilizer Development Center (IFDC) projects co-funded by the government of The Netherlands: 3500 topsoil samples (0–20 cm) for Uganda, Rwanda and Burundi also analyzed using soil spectroscopy. Majority of samples was collected in the period 2009–2014.
- One Acre Fund: some 2400 topsoil samples (0–20 cm) for Uganda and Kenya, collected in the period 2010–2016.
- University of California, Davis: some 1800 topsoil samples (0–20 cm) for Kenya.
- VitalSigns: 1374 soil samples from Ghana, Rwanda, Tanzania and Uganda also analyzed using mid-infrared spectroscopy, collected in the period 2013–2016.

We focused on producing spatial predictions for the following 15 nutrients (all concentrations are expressed as mass fractions using mg per kg soil fine earth i.e. ppm): organic carbon (C) and total nitrogen (N), total phosphorus (P), and extractable: phosphorus (P), potassium (K), calcium (Ca), magnesium (Mg), sulfur (S), sodium (Na), iron (Fe), manganese (Mn), zinc (Zn), copper (Cu), aluminum (Al) and boron (B). Although C, Na and Al are commonly not classified as soil nutrients, their spatial distribution can help assessment of soil nutrient constraints. For example, extractable Al can be an important indicator of soil production potential: high exchangeable Al levels can reduce growth of sensitive crops as soil pH (H_2_O) drops below <5.3 and become toxic to the majority of plants <4.5 (White 2009).

**Fig. 1:**
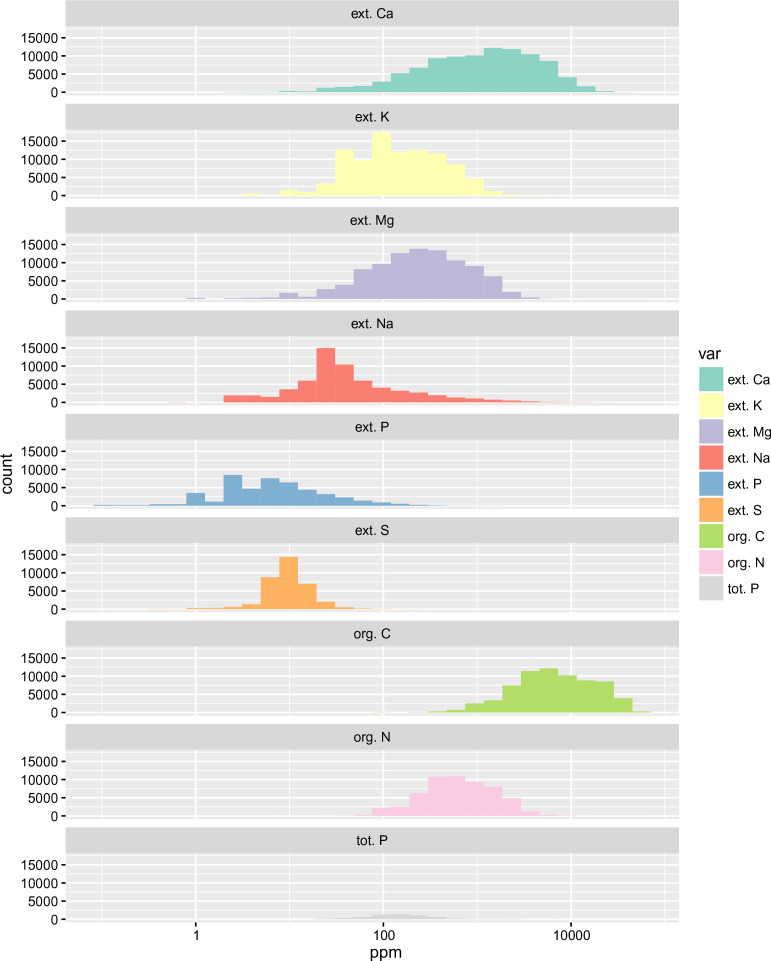
Combined histograms (at log-scale) for the soil macro-nutrients based on a compilation of soil samples for Sub-Saharan Africa

**Fig. 2:**
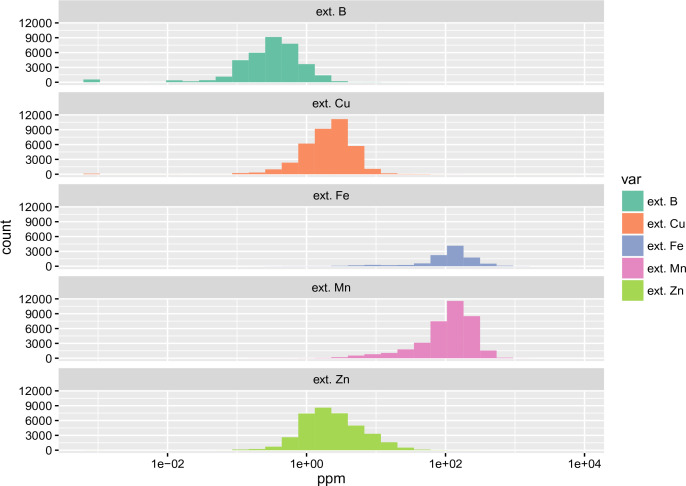
Combined histograms (at log-scale) for the soil micro-nutrients based on a compilation of soil samples for Sub-Saharan Africa

Histograms of nutrients based on the data compilation are depicted in Figs. 1 and 2. Although the soil data sources used for model calibration are quite diverse, the majority of soil samples had been analyzed using the MIR technology by the (same) soil-plant spectral diagnostics laboratory at the World Agroforestry Centre (ICRAF), Nairobi, and Crop Nutrition Laboratory Services, Nairobi, and are hence highly compatible.

The time span of field data collection is wide and legacy soil data points have diverse origins, often referring to field work done in the last 20+ years (1980–2016). Apart from the legacy soil profile data set, all other soil samples (>60%) are relatively up-to-date and refer to the period 2008–2016. The following two assumptions, therefore, must be borne in mind:

- the produced spatial predictions presented in this paper might not everywhere reflect current status of nutrients on the field, i.e. they should only be used as long-term, average estimates, and
- the temporal variation in soil nutrients is ignored—or in other words, dynamics of soil nutrients over the 1980–2016 span is not discussed in this work.

Note also that nutrient status, in terms of total amount of extractable nutrients (kg/ha) in the soil, is only partially reflected by relative nutrient contents (g/kg) in a limited depth interval of e.g. 0–20 cm. Thus the available amount of nutrients is only a fraction of the measured amount. Additionally, bulk density would be necessary for conversion to kg/ha. Regardless, concentrations are still highly relevant as most fertilizer recommendations are based on nutrient concentrations, rather than nutrient stocks.

**Fig. 3:**
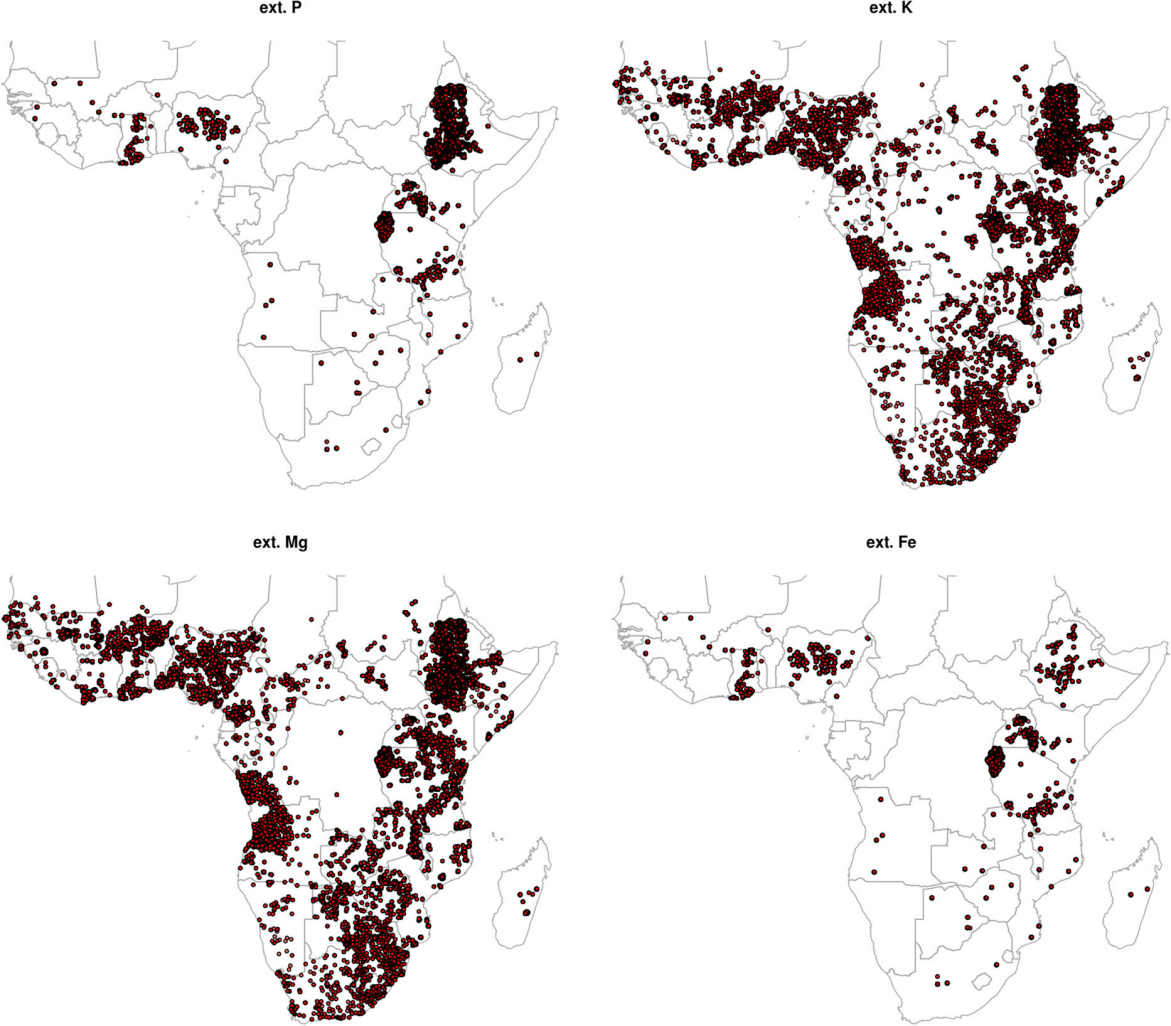
Comparison of spatial coverage of sampling locations for four nutrients: ext. P, ext. K, ext. Mg and ext. Fe. Data sources: AfSIS Sentinel Sites soil samples, EthioSIS soil samples, Africa Soil Profiles DB soil samples, IFDC-PBL soil samples, One Acre Fund soil samples, University of California soil samples and Vital Signs soil samples. See text for more details

Unfortunately, not all soil nutrients were available at all sampling locations. Figure 3 shows an example of the differences in the spatial spread of points for extr. P, K, Mg and Fe. For micro-nutrients such as Fe, it is obvious that points are spatially clustered and available only in selected countries. Large gaps in geographical coverage also often occur due to limitations on sampling such as accessibility and safety issues, so that especially tropical forests and wetlands are under-represented in the sampling designs. Nevertheless, in most of main sampling campaigns such as the AfSIS sentinel sites, locations were purposely selected to represent the main climatic zones (Vågen et al. 2010), so in this sense coverage of sampling locations can be considered satisfactory for most nutrients.

### Covariates

As spatial covariates, a large stack of GIS layers as proxies for soil forming processes (climate, landform, lithology and vegetation) was used:

- DEM-derived surfaces—slope, profile curvature, Multiresolution Index of Valley Bottom Flatness (VBF), deviation from mean elevation value, valley depth, negative and positive Topographic Openness and SAGA Wetness Index, all derived using SAGA GIS at 250 m resolution (Conrad et al. 2015);
- Long-term averaged monthly mean and standard deviation of the MODIS Enhanced Vegetation Index (EVI) at 250 m;
- Long-term averaged monthly mean and standard deviation of the MODIS land surface temperature (daytime and nighttime) based on the 1 km resolution data;
- Land cover map of the world at 300 m resolution for the year 2010 prepared by the European Space Agency (http://www.esa-landcover-cci.org/);
- Monthly precipitation images at 1 km spatial resolution based on the CHELSA climate data set obtained from http://chelsa-climate.org (Karger et al. 2016);
- Global cloud dynamics images at 1 km resolution obtained from http://www.earthenv.org/cloud (Wilson and Jetz 2016);
- Global cloud dynamics images at 1 km resolution obtained from http://www.earthenv.org/cloud (Wilson and Jetz 2016);
- Geologic age of surficial outcrops from the USGS map (at general scale) showing geology, oil and gas fields and geological provinces of Africa (Persits et al. 2002);
- Kernel density maps based on the Mineral Resources Data System (MRDS) points (McFaul et al. 2000), for mineral resources mentioning Fe, Cu, Mn, Mg, Al and Zn;
- Groundwater storage map, depth to groundwater and groundwater productivity map provided by the British Geological Survey (MacDonald et al. 2012);
- Landform classes (breaks/foothills, flat plains, high mountains/deep canyons, hills, low hills, low mountains, smooth plains) based on the USGS Map of Global Ecological Land Units (Sayre et al. 2014);
- Global Water Table Depth in meters based on Fan et al. (2013);
- Landsat bands red, NIR, SWIR1 and SWIR2 for years 2000 and 2014 based on the Global Forest Change 2000–2014 data v1.2 obtained from http://earthenginepartners.appspot.com/science-2013-global-forest (Hansen et al. 2013);
- Global Surface Water dynamics images: occurrence probability, surface water change, and water maximum extent (Pekel et al. 2016), obtained from https://global-surface-water.appspot.com/download;
- Distribution of Mangroves derived from Landsat images and described in Giri et al. (2011);
- Predicted soil pH (H_2_O) maps at 250 m produced within the SoilGrids project (https://soilgrids.org);

DEM derivatives were based on the global merge of SRTMGL3 DEM and GMTED2010 data sets (Danielson and Gesch 2011). Long-term estimates of EVI seasonality were derived using a stack of MOD13Q1 EVI images (Savtchenko et al. 2004); and long-term MODIS LST day-time and night-time images, also derived from a stack of MOD11A2 LST images (Wan 2006). Both MODIS products were based on data for the period 2000–2015. Global Surface Water dynamics images refers to period 1984–2015 and CHELSA climate images to period 1979–2015.

Remote sensing data had been previously downloaded and prepared via ISRIC’s massive storage server for the purpose of the SoilGrids project (Hengl et al. 2017). The majority of covariates cover the time period 2000–2015, i.e. they match the time span for most of the newly collected soil samples.

Prior to modeling, all covariates have been stacked to the same spatial grids of 250 m, as the best compromise between computational load and average resolution of all covariates. To downscale climatic images and similar coarser resolution images we used the bicubic spline algorithm as available in the GDAL software (Mitchell and Developers 2014).

### Spatial prediction framework

Model fitting and prediction were undertaken using an ensemble of two Machine Learning algorithms (MLA) (Hengl et al. 2017): ranger (random forest) (Wright and Ziegler 2016) and xgboost (Gradient Boosting Tree) (Chen and Guestrin 2016), as implemented in the R environment for statistical computing. Both random forest and gradient boosting have already proven to be efficient in predicting soil chemical and physical soil properties at the continental and global scale (Hengl et al. 2017). Packages ranger and xgboost were selected also because both are highly suitable for dealing with large data sets and support parallel computing in R.

For all target variables we use depth as a covariate, so that the resulting models make depth-specific predictions of target variables:

$$Y\left(xyd\right)=d+X_{1}\left(xy\right)+X_{2}\left(xy\right)+...+X_{p}\left(xy\right)$$ (1)

where *Y* is the target variable, usually nutrient concentration in ppm, *d* is the depth of observation and *X_p_*(*xy*) are covariates and *xy* are easting and northing. Note that there is somewhat bias in sampling representation towards top-soil as large portion of samples only has values for 0–20 cm depths i.e. represent only one depth. On the other hand, almost all of legacy soil profiles (18,500 locations) contain measurements for all horizons, so that building of soil variable-depth relationship is still possible.

We make predictions at four standard depths: 0, 5, 15, and 30 cm (at point support), and aggregate these to the 0–30 cm standard depth interval using the trapezoidal rule for numerical integration:

$$\int_{a}^{b}f\left(x\right)dx\approx \frac{1}{2}\sum_{k=1}^{N-1}\left(x_{k+1}-x_{k}\right)\left(f\left(x_{k+1}\right)+f\left(x_{k}\right)\right)$$ (2)

where *N* is the number of depths in the [*a,b*] interval where predictions were made, *x* is depth (*a* = *x*_1_<*x*_2_ <…<*x_N_* = b) and *f*(*x*) is the value of nutrient content at depth *x*. Although we could have made predictions for each 1 cm, for practical reasons (computational intensity and storage) four depths were considered good-enough to represent soil variable-per-depth changes. Depths 0, 5, 15, and 30 cm were chosen as standard depths also because these are standardly used in the SoilGrids project (Hengl et al. 2017). For several soil nutrients, especially organically bound nutrients as Nitrogen, Carbon, Sodium and to a lesser extent Phosphorus, modeling soil variable-depth relationship is important because the concentrations generally show distinct changes with depth.

We initially considered running kriging of remaining residuals, but eventually this was not finally considered worth the effort for the following two reasons. First, most of the observation points are far apart so kriging would have had little effect on the output predictions. Second, the variograms of the residuals all had a nugget-sill ratio close to 1, meaning that the residual variation lacked spatial structure and would not benefit spatial interpolation, e.g. by the use of kriging (Hengl et al. 2007). From our work so far on this and other soil related projects, it seems that there is a rule of thumb where once a machine learning model explains over 60% of variation in data, chances are that kriging is not worth the computational effort.

To optimize fine-tuning of the Machine Learning model parameters, the caret::train function (Kuhn 2008) was used consistently with all nutrients. This helped especially with fine-tuning of the xgboost model parameters and the mtry parameter used in the random forest models. Optimization and fine-tuning of Machine Learning algorithms was computationally demanding and hence time consuming, but our experience was that it often led to a 5–15% improvement in the overall accuracy.

All processing steps and data conversion and visualization functions have been documented via ISRIC’s institutional github account^2^. Access to legacy data from the Africa soil profiles database and other data sets produced by the AfSIS project is public and to access the other data sets consider contacting the corresponding agencies.

### Accuracy assessment

**Fig. 9:**
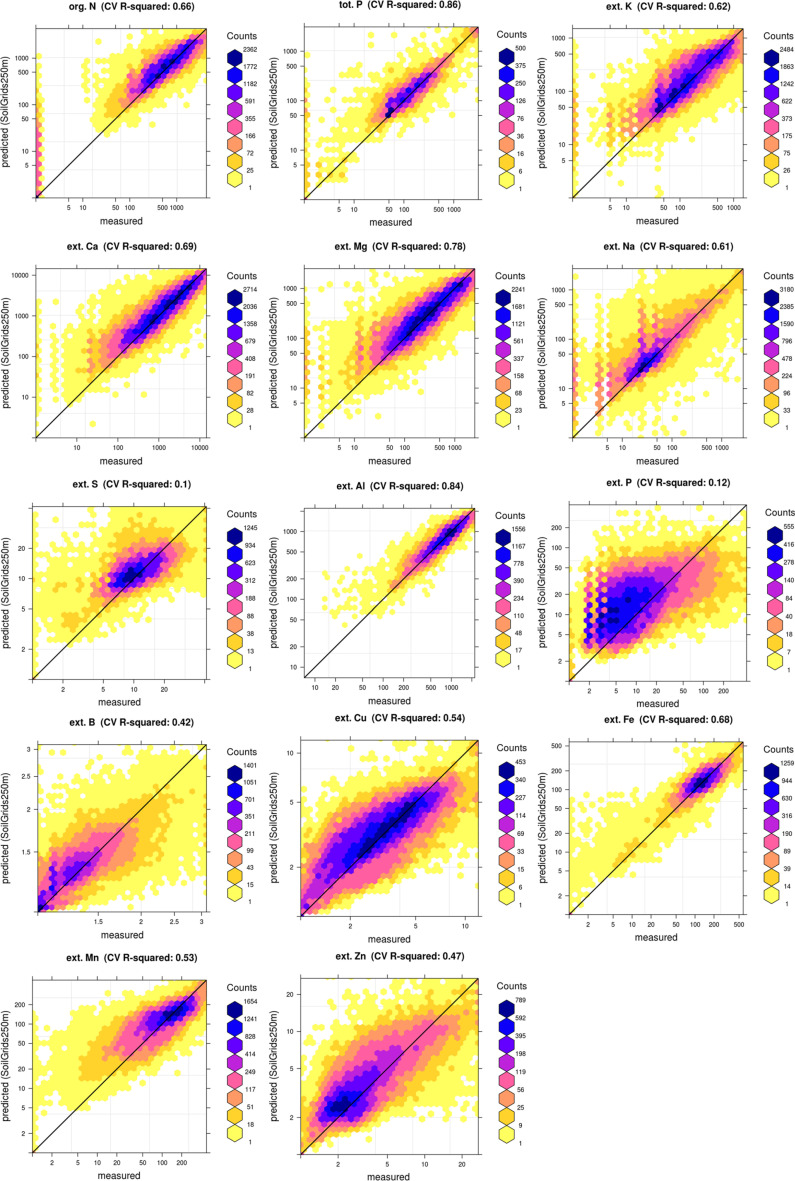
Accuracy assessment plots for all nutrients. Predictions derived using 5–fold cross-validation. All values expressed in ppm and displayed on a log-scale

For accuracy assessment a 5–fold cross-validation was used where each model was re-fitted five times using 80% of the data and used to predict at the remaining 20% (Kuhn 2008). Predictions were then compared with the put-aside observations. For each soil nutrient content, the coefficient of determination (*R^2^* , the amount of variation explained by the model) and root mean squared error (RMSE) was derived. The amount of variation explained by the model was derived as:

$$\sum_{\%}=\left[1-\frac{SSE}{SST}\right]=\left[1-\frac{RMSE^{2}}{\sigma_{z}^{2}}\right]\left[0-100\%\right]$$ (3)

where *SSE* is the sum of squares of residuals at cross-validation points (i.e. *RMSE^2^* · *n*), and *SST* is the total where *K* is the number of clusters, log*_K_* is the logarithm sum of squares. A coefficient of determination close to 1 indicates a perfect model, i.e. 100% of variation is explained by the model. As all soil nutrients had a near log-normal distribution, we report the amount of variation explained by the model after log-transformation. Also, for the cross-validation correlation plots (observed vs predicted; see further Fig. 9) log scale was also used to ensure equal emphasis on low and high values.

### Multivariate and cluster analysis

In addition to fitting models per nutrient, we also run multivariate and cluster analysis to determine cross-correlations and groupings in the values. First, we analyzed correlation between the nutrients by running principal component analysis. Secondly, we allocated individual sampling locations to clusters using unsupervised classification to determine areas with relatively homogeneous concentrations of nutrients. For this we used the fuzzy *k*-means algorithm as implemented in the h2o package (Aiello et al. 2016).

Both principal component analysis and unsupervised fuzzy *k*-means clustering were run on transformed variables using the Aitchison compositions as implemented in the compositions package (van den Boogaart and Tolosana-Delgado 2008). Note that transforming the original nutrient values into compositions is important as in its absence, application of statistical methods assuming free Euclidean space (e.g. PCA and unsupervised fuzzy *k*-means clustering) gives a highly skewed view of the variable space (van den Boogaart and Tolosana-Delgado 2008).

**Fig. 10:**
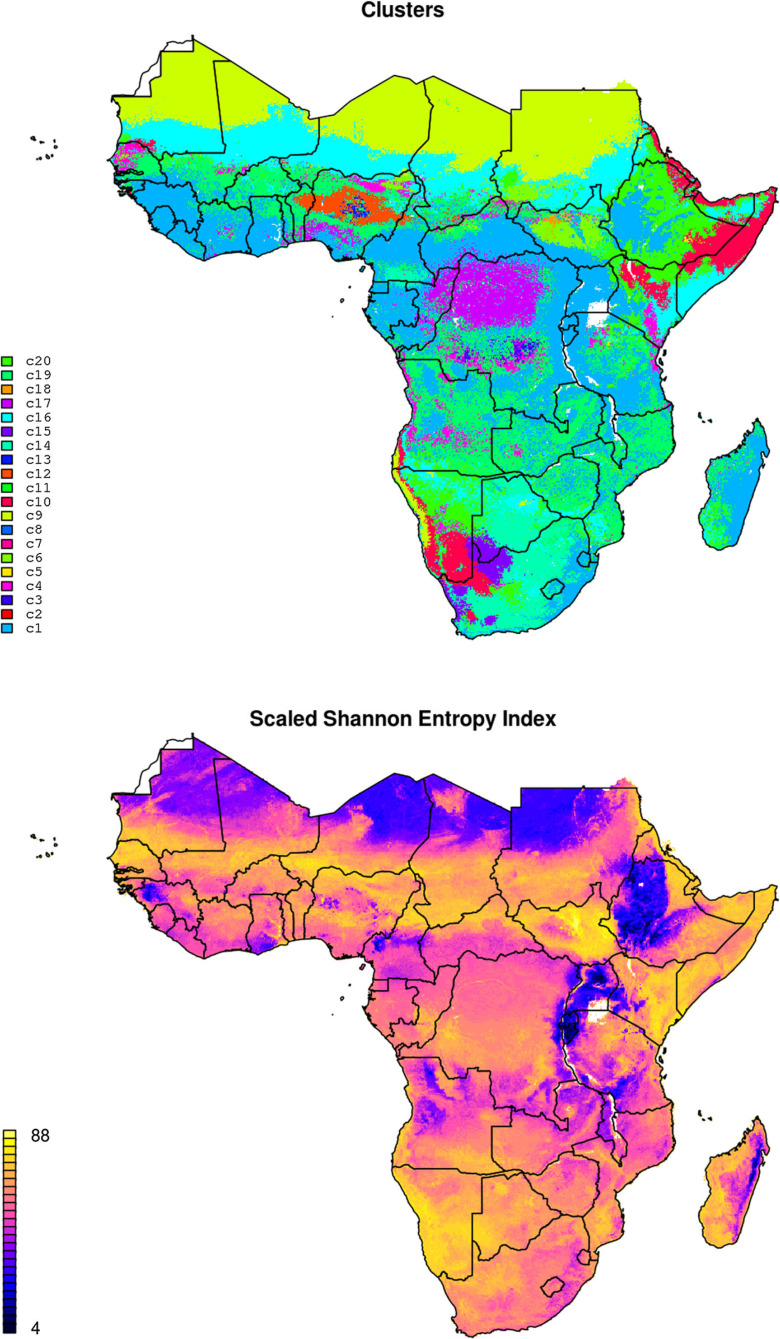
Predicted spatial distribution of the determined clusters (20) (*above*), and the corresponding map of scaled Shannon Entropy Index (*below*). High values in scaled Shannon Entropy Index indicate higher prediction uncertainty. Cluster centers are given in Table 3

After clusters in nutrient values were determined, they were correlated with the same stack of covariates used to model individual nutrients, and a random forest classification model was fit and used to generate predictions for the whole of SSA (see further Fig. 10). As probability maps were produced for each cluster, we also calculated a map of the Scaled Shannon Entropy Index (SSEI) to provide a measure of the classification uncertainty (Shannon 1949; Borda 2011):

$$H_{s}\left(x\right)=-\sum_{k=1}^{K}p_{k}\left(x\right)\cdot \log_{k}\left(p_{k}\left(x\right)\right)$$ (4)

where *K* is the number of clusters, log*_K_* is the logarithm to base *K* and *p_k_* is the probability of cluster *k*. The scaled Shannon Entropy Index (H*_s_*) is in the range from 0–100%, where 0 indicates no ambiguity (one of the *p_k_* equals one and all others are zero) and 100% indicates maximum confusion (all *p_k_* equal $\frac{1}{k}$) The H*_s_* indicates where the ‘*true*’ cluster is most uncertain.

In summary, the process of generating maps of nutrient clusters consists of five major steps:

1. Transform all nutrient values from ppm’s to compositions using the compositions package (van den Boogaart and Tolosana-Delgado 2008).
2. Determine the optimal number of classes for clustering using the mclust package (Fraley et al. 2012) i.e. by using the Bayesian Information Criterion for expectation-maximization.
3. Allocate sampling points to clusters using unsupervised classification with fuzzy *k*-means using the h2o package (Aiello et al. 2016).
4. Fit a spatial prediction model using the ranger package based on clusters at sampling points and the same stack of covariates used to predict nutrients.
5. Predict clusters over the whole area of interest and produce probabilities per cluster.
6. Derive scaled Shannon Entropy Index (SSEI) map and use it to quantify spatial prediction uncertainty.

### Importance of soil nutrient maps for crop yield data

In order to evaluate the importance of these soil nutrient maps for actual agricultural planning, we use the publicly available Optimising Fertilizer Recommendations in Africa (OFRA) field trials database. OFRA, a project led by CABI (Kaizzi et al. 2017), contains 7954 legacy rows from over 600 trials collected in the period 1960–2010. Field trials include crop yields and field conditions for majority of crops including maize, cowpea, sorghum, (lowland, upland) rice, groundnut, bean, millet, soybean, wheat, cassava, pea, climbing bean, barley, sunflower, (sweet, irish and common) potato, cotton, and similar. The OFRA database covers only 13 countries in Sub-Saharan Africa, hence it does not have the ideal representation considering all combinations of climatic and land conditions of crop growing. It is, nevertheless, the most extensive field trial database publicly available for SSA.

We model the relationship between the crop yield and mapped climatic conditions (monthly temperatures and rainfall based on the CHELSA data set), mapped soil nutrients using a single model in the form:

$$cropyield\left[t/ha\right]=f\left\{croptype,\mathrm{variety},application,nutrients,climate\right\}$$ (5)

where cropyield, croptype, variety and application are defined in the OFRA database, nutrients are maps we produced, and climate are CHELSA climatic images for SSA. Variable croptype is the factor type variable (e.g. ‘‘maize’’, ‘‘cowpea’’, ‘‘sorghum’’, ‘‘rice’’ etc) and so are variety (e.g. ‘‘H625’’, ‘‘Glp 2’’, ‘‘Maksoy2&4’’ etc) and application (‘‘2 splits’’, ‘‘2/3 applied basally’’, ‘‘all fertilizer applied along the furrows’’ etc). This model we also fit using random forest, so that crop yields can be differentiated for various crop types, covariates and crop applications via a single model. Once the model in Eq. (5) is fitted, it can be used to generate predictions for various combinations of the former, which could lead to an almost infinite number of possible maps.

Here we primarily concentrate on testing whether soil nutrients are important factor controlling crop yield. Note also that fitting one model for all crop types is statistically elegant (one multivariate model to explain all crop yield) also because one can then explore all interactions e.g. between crop types, varieties, treatments etc, and produce predictions for all combinations of crop types, varieties and treatments; this would have been otherwise very difficult if not impossible if we were to fit models per each crop type.

## Results

### Principal component analysis

**Fig. 4:**
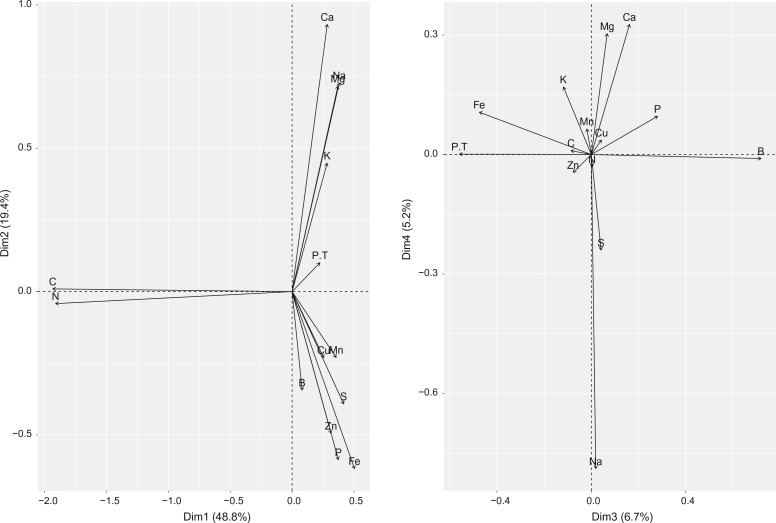
Principal component analysis plots generated using sampled data: (*left*) biplot using first two components, (*right*) biplot using the third and fourth component. Prior to PCA, original values were transformed to compositions using the compositions package. P is the extractable phosphorus, and P.T is the total phosphorus

The results of the principal component analysis (Fig. 4) shows that there are two positively correlated groups of nutrients (K, Mg, Na and Ca and org. C and N and total P). Negatively correlated nutrients are: Na, Mg, Ca vs Fe, Zn, Cu, Mn and S (i.e. high iron content commonly results in low Na, Mg, Ca content), and B vs org. C and N and total P. Because most nutrients were inter-correlated, >75% of variation in values can be explained with the first five components: PC1 (48.8 %), PC2 (19.4%), PC3 (6.7%), PC4 (5.2%) and PC5 (3.8% variation).

### Model fitting results

The model fitting and cross-validation results are shown in Table 1 and most important covariates per nutrient are shown in Table 2. For most of nutrients successful models can be fitted using the current set of covariates with R-square at cross-validation points ranging from 0.40 to 0.85. For extractable S and P, models are significantly less prominent and hence the maps produced using these models can be associated with wide prediction uncertainty (hence probably not ready for operational mapping).

**Table 1:** List of target soil macro- and micro-nutrients of interest and summary results of model fitting and cross-validation

Nutrient	Method	N	1%	50%	99%	R-square	RMSE
org. N	total (organic) N extractable by wet oxidation	63,937	0.0	600.0	4200	0.66	558
tot. P	total phosphorus	7899	0	132	3047	0.85	284
ext. K	extractable by Mehlich 3	104,784	0	130	1407.5	0.64	201
ext. Ca	extractable by Mehlich 3	105,173	14	1162	14288	0.69	1950
ext. Mg	extractable by Mehlich 3	103,356	1.2	242	2437	0.78	241
ext. Na	extractable by Mehlich 3	71,986	0	30.13	2690	0.61	452
ext. S	extractable by Mehlich 3	43,666	0.6	9	51	0.11	78
ext. Al	extractable by Mehlich 3	30,945	0	874	2120	0.84	171
ext. P	extractable by Mehlich 3	42,984	0	6	188	0.12	43
ext. B	extractable by Mehlich 3	43,338	0	0.33	2.09	0.41	0.47
ext. Cu	extractable by Mehlich 3	45,572	0.001	2.2	10.6	0.54	2.11
ext. Fe	extractable by Mehlich 3	18,341	0	121	574	0.68	53
ext. Mn	extractable by Mehlich 3	44,689	1.8	124	440	0.53	69
ext. Zn	extractable by Mehlich 3	45,626	0.1	2.1	26.03	0.47	4.0

All values are expressed in ppm. N = ‘‘Number of samples used for training’’, R-square = ‘‘Coefficient of determination’’ (amount of variation explained by the model based on cross-validation) and RMSE = ‘‘Root Mean Square Error’’. Underlined cells indicate poorer models (or too small sample sizes)

**Table 2:** Top ten most important covariates per nutrient, reported by the ranger package

Nutrient	Most important covariates (10)
org. N	Depth, LSTD November, TWI (DEM), LSTD October, precipitation November, soil pH, DEM, water vapor January-February, precipitation December, mean annual temperature
tot. P	Precipitation July, density of mineral exploration sites (Al), precipitation August, September, lithology, precipitation February, LSTD August, mean annual precipitation, water vapor January-February, precipitation June
ext. K	Soil pH, water vapour July-August, DEM, precipitation January, std. EVI April, precipitation February, water vapor January-February, depth, cloud fraction February, water vapor November-December
ext. Ca	Soil pH, water vapour January-February, water vapour November-December, cloud fraction March, DEM, mean EVI May-June, Landsat NIR, std. LSTD November, mean EVI July-August, Landsat SWIR1
ext. Mg	Soil pH, water vapor January-February, Landsat NIR, Landsat SWIR1, cloud fraction February, Landsat SWIR2, water vapor November-December, LSTD March, water vapor March-April, Landsat SWIR1
ext. Na	Soil pH, depth, cloud fraction seasonality, cloud fraction March, LSTN December, mean EVI January-February, slope (DEM), std. LSTN April, mean EVI May-June, LSTD July
ext. S	Lithology, Landsat SWIR2, cloud fraction December, precipitation October, May, TWI (DEM), precipitation November, std. EVI July-August, LSTD November
ext. Al	Soil pH, LSTD November, precipitation November, TWI, LSTD December, cloud fraction November, DEM, cloud fraction December, precipitation total, precipitation February
ext. P	Valley depth (DEM), precipitation July, Deviation from mean (DEM), precipitation November, DEM, std. EVI May-June, precipitation January, positive openness (DEM), mean EVI July-August, mean EVI May-June
ext. B	Precipitation August, January, depth, precipitation November, soil pH, DEM, std. EVI July-August, precipitation September, positive openness (DEM), precipitation December
ext. Cu	Water vapor May-June, precipitation December, water vapor November-December, July-August, September-October, depth, water vapor January-February, precipitation July, cloud fraction November, precipitation August
ext. Fe	Water vapor January-February, density of mineral exploration sites (Phosphates), water vapor September-October, July-August, cloud fraction seasonality, water vapor May-June, March-April, depth, DEM, cloud fraction mean annual
ext. Mn	Depth, precipitation November, April, cloud fraction January, land cover, DEM, precipitation February, January, water vapor January-February, precipitation December
ext. Zn	Precipitation January, December, mean EVI May-June, precipitation March, std. EVI March-April, precipitation February, November, April, TWI

Explanation of codes: depth = depth from soil surface, LSTD = MODIS mean monthly Land Surface Temperature day-time, LSTN= MODIS mean monthly Land Surface Temperature night-time, EVI = MODIS Enhanced Vegetation Index, TWI = topographicwetness index, DEM = Digital Elevation Model, NIR = Landsat Near Infrared band, SWIR = Landsat Shortwave Infrared band.Underlined covariates indicate distinct importance

The model fitting results show that the most important predictors of soil nutrients are usually soil pH, climatic variables (precipitation and temperature), MODIS EVI signatures and water vapor images. The order of importance varies from nutrient to nutrient: soil pH is clearly most important covariate for Na, K, Ca, Mg, Al; it is somewhat less important for N. Precipitation (especially for months November, December and January) distinctly comes as the most important covariate overall. Considering that soil pH, at global scale, is mostly correlated with precipitation (Hengl et al. 2017), this basically indicates that precipitation, overall, comes as the most important covariate.

The fact that Landsat bands also come as important covariate for number of nutrients (Na, Ca, Mg, S) is a promising discovery for those requiring higher resolution maps (Landsat bands are available at resolution of 60–30 m). Nevertheless, for majority of nutrients, the most important covariates are various climatic images, especially precipitation images. Although climatic images are only available at coarse resolution of 1 km or coarser, it seems that climate is the key factor controlling formation and evolution of nutrients in soil.

Model fitting results also show that apart from org. C and N, and ext. Mn, Fe, B and P, the majority of nutrient values do not change significantly with depth. For the majority of soil macro- and micro-nutrients, it is probably enough to sample nutrients at a single depth. For C, N, P, Mn, Fe and B, depth is relatively high on the list of important covariates and hence should not be ignored.

### Spatial predictions

**Fig. 5:**
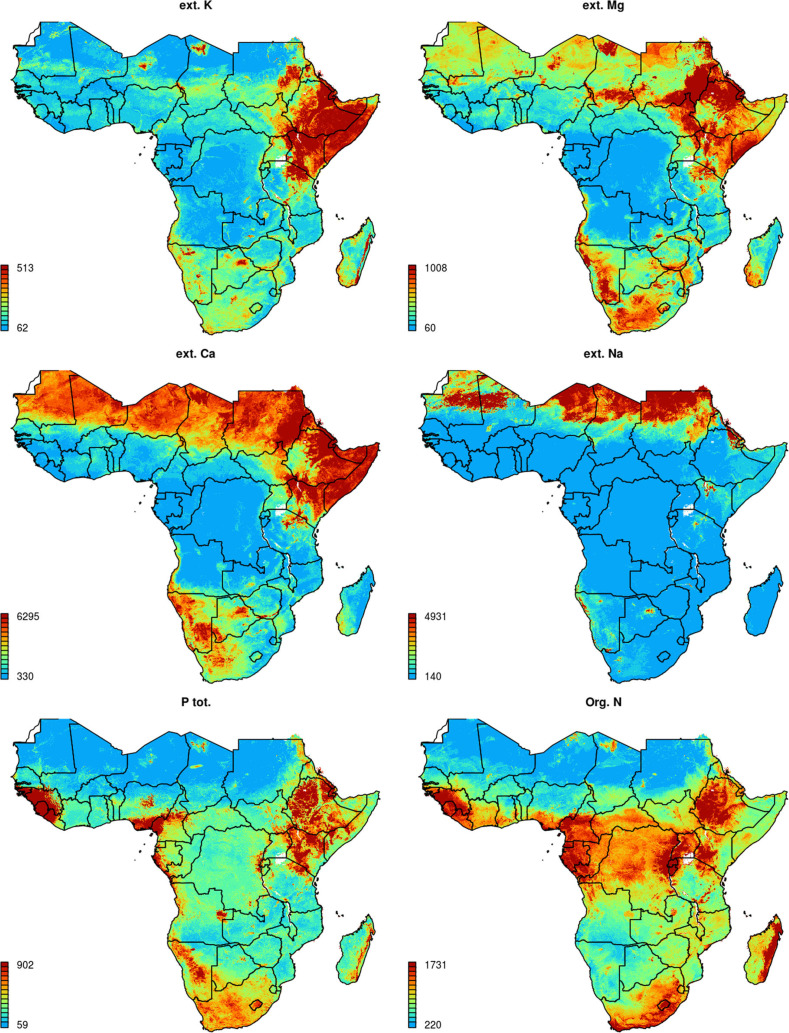
Predicted soil macro-nutrient concentrations (0–30 cm) for Sub-Saharan Africa. All values are expressed in ppm

**Fig. 6:**
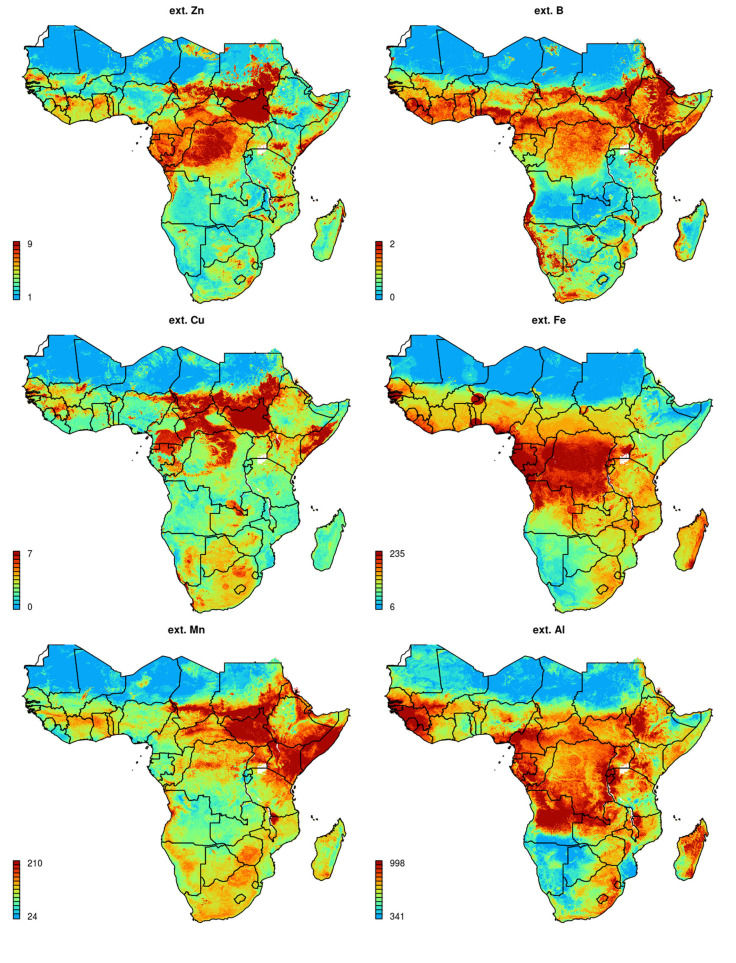
Predicted soil micro-nutrient concentrations (0–3 cm) and extractable Al for Sub-Saharan Africa. All values are expressed in ppm

Final spatial predictions for nutrients with significant models are shown in Figs. 5 and 6. The spatial patterns produced match our expert knowledge and previously mapped soil classes in general, which is true especially for Fe, org. C and N and Ca and Na. Our predictions also indicate that the highest deficiencies for B and Cu are in sub-humid zones, which corresponds to the results of Kang and Osiname (1985). As several of the micro-nutrients have been mapped for the first time for the whole of Sub-Saharan Africa, many produced spatial patterns will need to be validation both locally and regionally.

Some artifacts, in the form of sharp gradients that could not occur naturally, are visible in the output maps, primarily due to the very coarse resolution of the geological layer used for model building. Unfortunately, the lithological map (Persits et al. 2002) and the map of groundwater resources (MacDonald et al. 2012), were available only at relatively general scale, i.e. these corresponds to spatial resolutions of 10 km or coarser, so that consequently artifacts, due to resolution mismatch and manually drawn geomorphological boundaries, are also visible in the output predictions.

Predictions of all soil nutrients at four depths took approximately 40 h on ISRIC’s dedicated server with 256 GiB RAM and 48 cores (whole of Sub-Saharan Africa is about 7500 by 7000 km, i.e. covers about 23.6 million square kilometers). Fitting of models on the dedicated server running R software is efficient and models can be generated within 1 hour even though there were, on average, >50,000 of measurements per nutrient. With some minor additional investments in computing infrastructure, spatial predictions could be updated in future within 24 hrs (assuming all covariates are ready and harmonization of nutrients data already implemented).

**Fig. 7:**
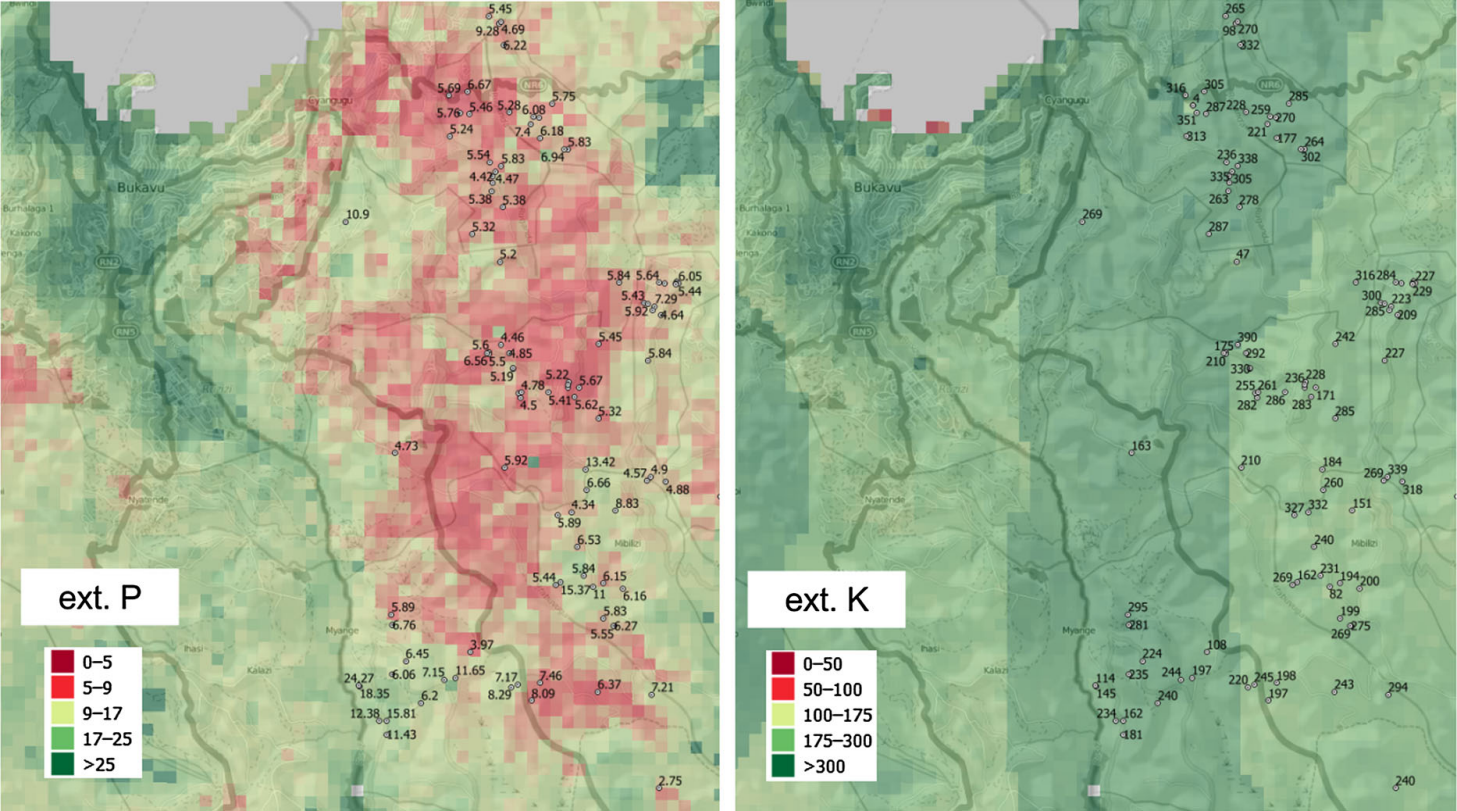
Examples of nutrient deficiency maps based on our results: zoom in on town Bukavu at the border between the eastern Democratic Republic of the Congo (DRC) and Rwanda. *Points* indicated samples used for model training. The thresholdlevels are based on Roy et al. (2006, p.78) ranging from very low (<50% expected yield) to medium (80–100% yield) to very high (100% yield). All values are in ppm’s. Background data source: OpenStreetMap

**Fig. 8:**
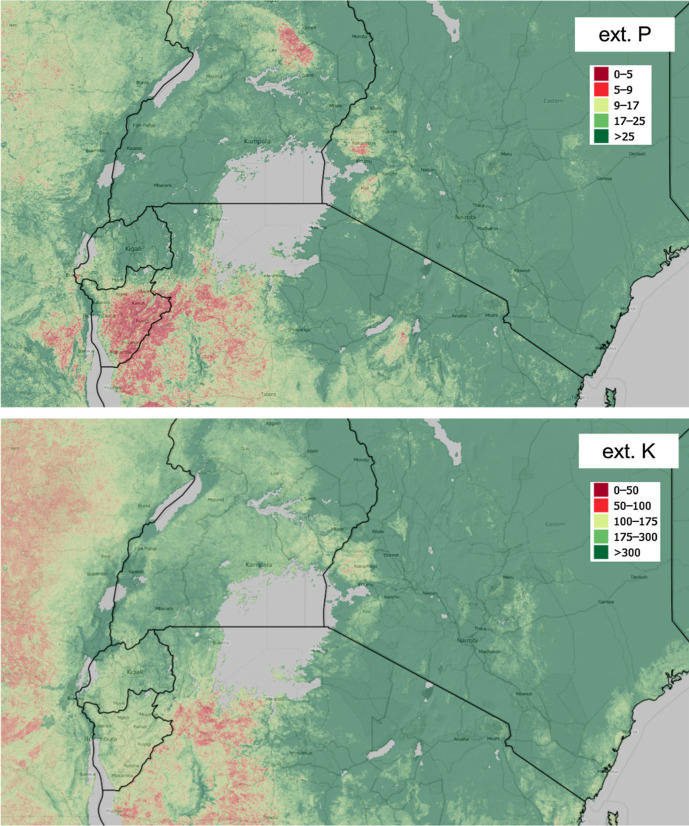
Examples of locally defined nutrient deficiency maps based on our results: Eastern Africa. The adopted threshold levels are based on Roy et al. (2006, p.78) ranging from very low (<50% expected yield) to medium (80–100% yield) to very high (100% yield). All values are in ppm’s. Background data source: OpenStreetMap

Figures 7 and 8 show the level of spatial detail of the output maps and demonstrates how these maps could be used for delineation of areas potentially deficient in key soil nutrients, i.e. a somewhat more useful/interpretable form of summary information to agronomists and ecologists. In this case, determination of deficient and suitable nutrient content was based on soil fertility classes by Roy et al. (2006), ranging from very low (>50% expected yield) to medium (80–100% yield) to very high (100% yield) and assuming soil of medium CEC. Crop specific threshold levels can be set by users to quickly map areas of nutrient deficiency/high potential fertility to spatially target suitable agronomic intervention. Similar, threshold values beyond which crop does not respond to fertilizer nutrient application can be diversified and mapped at regional scale based on the spatial diversity of measured or calculated attainable yield levels.

### Accuracy assessment results

The cross-validation results are reported in Table 1and in Fig. 9. For org. C and N, extractable K, Ca, Na, Mg, Fe, Mn, Cu and Al, cross-validation R-square wasabove 50%, which is often considered a solid result insoil mapping projects (Hengl et al. 2015). For S and ext. P we could not fit highly significant models (R-square <30%). It could be very difficult, if notimpossible, to make any significant maps of estimatesof extractable soil P and S with the existing data, hencethese maps should be used with caution. However, maps of other nutrients and also of properties such as pH and CEC could be useful for informing the potential for low contents in these elements, for example association of high P fixation and low extractable P with high Fe and Mn, and low sulfur in soils with low C.

### Spatial distribution of soil nutrient clusters

The results of the cluster analysis show that the optimal number of clusters, based on the Bayesian Information Criterion for expectation-maximization, initialized by hierarchical clustering for parameterized Gaussian mixture models, as implemented in the mclust package function Mclust (Fraley et al. 2012), can be set at 20. It appears, however, that optimal number of clusters cannot be set clearly as majority of points were not split into distinct clouds, hence other smaller and larger numbers than 20 could have been derived probably with other similar cluster analysis packages.

A random forest model fitted using 20 clusters shows significance with an average out-of-bag classification accuracy, reported by the ranger package, of 65%. Class centers and corresponding interpretations are shown in Table 3, while the spatial distribution of clusters of soil nutrients is shown in Fig. 10. Note that, although it might seem difficult to assign meaningful names to clusters, it is clear that for example cluster c1 can be associated with high organic C and N, and cluster c11 with high K content. Cluster analysis shows that especially classes 8, 12 and 13 have systematic deficiencies in most of micro-nutrients; classes 2 and 7 shows specific nutrient deficiencies in K and Mg.

**Table 3:** Class centers for 20 clusters determined using supervised fuzzy *k*-means clustering

Cluster	org. C	org. N	K	P	P tot.	Ca	Mg	Na	S	Fe	Mn	Zn	Cu	B
c1	23,400	1680	247	13.7	874	1570	306	113	13	123	119	3.8	2.2	0.4
c2	1840	280	*54.7*	12.4	344	321	*56*	*0*	49	97	250	47.0	60.0	0.7
c3	2230	286	115	78.7	366	463	114	48	29	134	128	5.3	2.7	0.9
c4	3580	335	109	33.7	333	631	162	69	23	117	120	4.7	2.8	0.6
c5	3090	383	211	27.9	449	1720	282	171	626	99	134	4.2	2.7	2.4
c6	1890	246	76.3	13.4	*163*	628	166	76	24	86	130	9.5	5.4	0.4
c7	1190	291	*58*	19.5	237	295	*95*	46	28	115	121	4.7	2.3	2.6
c8	*1*	*0*	62	*7.55*	290	231	104	36	9	193	*55*	*1.3*	1.7	*0.1*
c9	2840	372	335	21.3	303	3780	669	4270	56	*69*	116	4.1	2.5	0.7
c10	3870	439	297	27	444	3200	416	267	246	90	166	4.1	2.9	2.1
c11	5780	704	1740	34.4	607	2840	572	474	33	86	117	4.4	2.6	0.9
c12	*4*	*1*	112	7.74	278	797	214	41	*8*	140	74	1.5	*1.1*	*0.1*
c13	34	5	79.4	*4.36*	821	447	133	37	*8*	114	*46*	*1.1*	*0.9*	*0.1*
c14	3750	803	101	22.7	465	518	150	59	21	116	113	4.0	2.6	0.6
c15	1620	1040	82.2	26.6	482	332	115	56	28	108	116	3.9	2.8	0.6
c16	4260	514	269	21.8	451	5790	1180	496	41	76	136	4.3	3.2	0.8
c17	3200	393	65.6	21.6	301	357	127	51	22	139	122	5.0	2.4	1.8
c18	2600	330	64.7	15	*179*	742	145	*25*	51	101	330	27.5	30.1	0.6
c19	6890	580	133	24.4	413	665	162	56	17	122	114	4.0	2.3	0.5
c20	13,700	1100	416	20.8	756	5820	944	262	17	*67*	132	2.3	3.2	0.7

Underlined numbers indicate highest values per nutrient; italic indicates top two lowest concentrations per class

Map in Fig. 10 confirms that the produced clusters, in general, match combinations of climate and lithology. A map of the scaled Shannon Entropy Index (SSEI) for produced clusters is also shown in Fig. 10 (right). The differences in uncertainty for different parts of Sub-Saharan Africa are high. Especially large parts of Namibia, Democratic Republic Congo, Botswana, Somalia and Kenya have relatively high scaled Shannon Entropy Index (SSEI), hence higher uncertainty. In general it can be said that the SSEI map closely corresponds to extrapolation effects, i.e. that uncertainty primarily reflects density of points—as we get further away from main sampling locations, the SSEI grows to >80% (high uncertainty). In that sense, further soil sampling campaigns, especially in areas where the SSEI is >80%, could help decrease uncertainty of mapping soil nutrients in Sub-Saharan Africa. Map of SSEI is provided via the download data section.

### Correlation with crop yield data

**Fig. 11:**
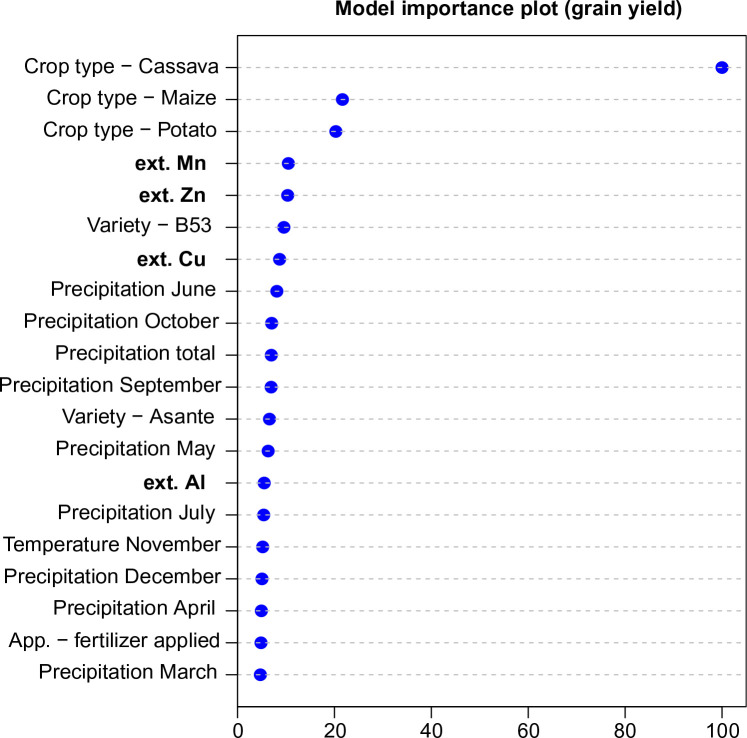
Variable importance plot for prediction of the crop yield using the model from Eq. (5). Training points include 7954 legacy rows for 606 trials

The result of modeling relationship between crop yield and nutrient and climatic maps (Eq. 5) show that a potentially accurate model can be fitted using random forest: this model explains 78% of variation in the crop yield values with an Out-Of-Bag (OOB) RMSE of ±2:4 t ha^−1^ . The variable importance plot (Fig. 11) further shows that the most influential predictors of the crop yield are: crop type, selection of nutrients and micro-nutrients (Mn, Zn, Al, B, Na), and, from climatic data, primarily monthly rainfall for June, October, September, May and July. This proves that producing maps of soil nutrients is indeed valuable for modeling agricultural productivity.

Note however that, although some micro-nutrients such as Mn and Zn come highest on the variable importance list, this does not necessarily makes them the most important nutrients in Africa. Figure 11 only indicates that these nutrients matter the most for the crop yield estimated at OFRA points. Also note that because soil nutrients are heavily cross-correlated (Fig. 4) relatively high importance of Mn and Zn could also indicate high importance of P, B, Fe and/or S.

**Fig. 12:**
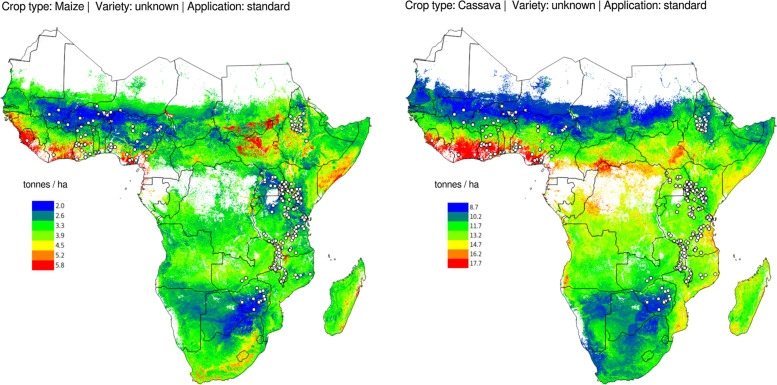
Examples of predicted potential crop yield for the land mask of SSA (excluding: forests, semi-deserts and deserts, tropical jungles and wetlands). *Circles* indicate the OFRA field trials database points used to train the model

Again, although the potential yield modeling results are promising and although even maps of potential crop yield can be generated (Fig. 12) using this model, we need to emphasize that these results should be taken with a reserve. Especially considering the following limitations:

1. Distribution of the OFRA field trials is clustered and limited to actual 606 trials/locations (Fig. 12), hence probably not representative of the whole SSA.
2. Most of field trials are legacy trials (often over 20 years old) and hence correlating them with the current soil conditions probably increases uncertainty in the models.
3. This model ignores weather conditions for specific years (instead long-term estimates of rainfall, temperatures are used). Matching exact weather conditions per year would probably be more appropriate.

If all OFRA training data was temporally referenced (day or at least month of the year known), we could have maybe produced even higher accuracy maps of potential crop yield e.g. by using the model of type:

$$cropyield\left(t/ha\right)=f\left\{{}_{nutrients\left(t\right),weather\left(t\right),av.water\left(t\right)}^{croptype\left(t\right),\mathrm{variety}\left(t\right),application\left(t\right),}\right\}$$ (6)

so that also very dry and very wet months and their impact during the growing season could be incorporated into the model. Unfortunately, temporal reference (begin/end date of application) in OFRA trials is often missing. Also weather maps for specific months for the African continent are only available at relatively coarse resolutions (e.g. 10 km or coarser) and often not available for periods before year 2000 at all.

## Discussion

In the following section we address some open issues and suggest the approaches to overcome these. This is mainly to emphasize limitations of this work, and to try to announce future research directions.

### Harmonization problems

One of the biggest problems of mapping soil nutrients for large areas are the laboratory and field measurement diversity. There is large complexity considering the methods and approaches to measurement of soil nutrients (Barber 1995). At farm-scale, this might not pose a too serious problem, but for pan-continental data modeling efforts it is certainly something that can not be ignored. In principle, many extractable soil nutrient content determination methods are highly correlated and harmonization of values is typically not a problem. For example, Phosphorus can be determined using Bray-1, Olsen and Mehlich-3 methods, which are all highly correlated depending on the pH range considered (Bray-1 and Mehlich-3 could be considered equivalent in fact). Conversion from one method to another however depends also on the soil conditions, such as soil pH and soil types (Roy et al. 2006) and requires data which are not readily available.

Some nutrient measurements might come from the X-ray fluorescence method (XRF), especially where plant available nutrient levels relate to total element concentrations (Towett et al. 2015). In this project, we did not invest in harmonization of measurement methods as this was well beyond the project budget. It is, for example, well known that extractable P, K and micro-nutrients do not predict well from MIR, hence there are still many limitations with using nutrient concentrations derived from soil spectroscopy. Improving harmonization, geolocation accuracy of samples and standardizing sufficiently large measurement support sizes (some samples were taken at fixed depths restricted to the topsoil, others were taken per soil horizon over soil depth), could possibly help improve accuracy of predictions.

### Computational challenges

Machine learning methods have already been proven effective in representing complex relationships with large stacks of variables (Strobl et al. 2009; Biau 2012). However, MLA’s can demand excessive computing time. Even though possibly more accurate, more generic algorithms than ranger and xgboost exist, these might require computing time which is beyond the feasibility of this project. For example, we have also tested using the bartMachine (bayesian additive regression trees) (Kapelner and Bleich 2013) and cubist (Kuhn et al. 2013) packages for generating spatial predictions, but due to very excessive computing times (even with full parallelization) we had no choice but to limit prediction modeling to ranger and xgboost. Computing time becomes a limiting factor especially as the number of training points is ≫10,000 and number of predictions locations goes beyond few million. In our case, whole of Sub-Saharan Africa at 250 m is an image of ca. 29,000 by 28,000 pixels, i.e. about 382 million pixels to represent the land mask.

### Critically poor predictions for P and S

Although the preliminary results presented in this paper are promising and many significant correlations have been detected, for nutrients such as ext. P, S and B we obtained relatively low accuracies. It could very well be that these types of nutrients will be very difficult to map at some significant accuracy using this mapping framework. To address these shortcomings in the near future one could test developing spatial predictions at high spatial detail e.g. at 100 m spatial resolution, and/or test developing spatiotemporal models for mapping the space-time dynamics of soil nutrients over Africa. Drechsel et al. (2001), for example, recognized that much of the soils of Sub-Saharan Africa are actually constantly degrading, hence spatiotemporal modeling of nutrients could probably lead to higher accuracy in many areas. In addition, all soil tests need calibration with crop response trials for different soil types and climates, and future efforts may be better directed at more accurate calibration of crop responses to soil test data.

Since this study focussed on predictions of soil nutrients using soil samples from a long period of years (1980–2016), we cannot tell from the current data what the rate of soil nutrient depletion is, nor where it is most serious. As nutrient contents can also be quite dynamic and controlled by the land use system (especially for nitrogen and organic carbon, and potentially phosphorus depending on fertilisation history), spatiotemporal models which take into account changes in land use could help increase mapping accuracy. Although we already have preliminary experience with developing spatiotemporal models for soil data, there are still many methodological challenges that need to be addressed, e.g. especially considering poor representation of time within the given sampling plans.

### Missing covariates

Accuracy of spatial predictions of nutrients could also be improved by investing in new and/or more detailed covariates. Unfortunately, no better parent material i.e. surface lithology map was available to us than the most general map of surface geology provided by USGS (Persits et al. 2002). Kang and Osiname (1985) suggests that the micro-nutrient deficiencies are especially connected with the type of parent material, hence lack of detailed parent material/lithology map of Africa is clearly a problem. Using gamma-radiometrics images in future could likely help increase accuracy of nutrient maps (especially for P and K). In Australia for example, a national agency has collected and publicly released gamma-radiometrics imagery for the whole of the continent (Minty et al. 2009); similar imagery is also available for the whole of conterminous USA (Duval et al. 2005). Although it is not realistic to expect that the African continent would soon have an equivalent, gamma-radiometric imagery could contribute substantially to regional soil nutrient mapping due to its ability to differentiate topsoil mineralogy. The recent initiatives such as the World Bank’s Sustainable Energy, Oil, Gas and Mining Unit (SEGOM) programme ‘‘*The Billion Dollar Map’’* (Ovadia 2015), could only help with bridging these gaps.

Another opportunity for increasing the accuracy of maps of nutrients is to try to utilize Landsat 8 and Sentinel-2 near and mid-infrared imagery to derive proxies of surface minerology. Several research groups are now working on integrating airborne/satellite sensing with ground-based soil sensing into a single framework (see e.g. work of Stevens et al. (2008) and Ben-Dor et al. (2009)). The newly launched SHALOM Hyperspectral Space Mission (Feingersh and Dor 2016) could be another source of possibly crucial remote sensing data for nutrient mapping and monitoring.

### Other soil nutrient data bases of interest

Accuracy and value of produced predictions could be improved if more sampling points were added to the training dataset, especially those funded and/or collected by national government agencies and NGO’s. Relevant data from additional soil data sets (not currently available for spatial prediction or with unknown user restrictions) include the AfSIS data recently generated in collaboration with Ethiopia, Tanzania, Ghana and Nigeria, ICRAF (World Agro-forestry Centre) and CIAT (The International Center for Tropical Agriculture) institutes additional data generated in collaborative projects, private sector funded data (e.g. MARS in Ivory Coast and others in Ivory Coast, Nigeria), USAID-funded IFDC project (https://ifdc.org/) data from West Africa, CASCAPE project (http://www.cascape.info/) data sets, N2Africa project samples (http://www.n2africa.org/), and data generated by various national initiatives.

As gradually more soil samples are added, especially in the (extrapolation) areas with highest spatial prediction error, it is reasonable to expect that the models and derived maps will also gradually become better. If not more accurate, then at least more representative of the main lithologic, climatic and land cover conditions in the SSA.

### Usability of produced maps

There is a critical need for agricultural and ecological data in Africa, where an expected 3.5–fold population increase this century (Gerland et al. 2014) will place immense demand on soil nutrients that form the basis of food production. Researchers and policy makers have repeatedly called for data and monitoring systems to track the state of the world’s agriculture (Sachs et al. 2010). In response to this need, this soil nutrients data set provides both a useful tool for researchers interested in the role that soil nutrients play in ecological, agricultural and social outcomes in Sub-Saharan Africa, as well as a general estimate of soil nutrient stocks at a time when the continent is facing significant climate and land-use change.

As the resolution of maps is relatively detailed, it is possible to spatially identify regional areas (Figs. 7, 8) that are ‘*naturally*’: (1) deficient, (2) adequate and (3) in excess relative to specific land-use requirements; and pair these with the nutrient-specific agronomic interventions required to achieve critical crop thresholds. Such usage could help optimize the use of soil resource and possibly (major) agronomic interventions across African countries (Vanlauwe et al. 2014). These agronomic interventions could consist of: targeting degraded areas that are suitable for restoration projects, and/or targeting areas for agricultural intensification and investment by modeling crop suitability and yield gaps at the regional scale (Nijbroek and Andelman 2016), and/or assessing the nutrient gaps to predict fertilizer nutrient use efficiency.

Although we have only estimated long-term nutrient contents using relatively scarce data, the maps produced could be used to derive various higher-level data products, such as nutrient mass balance maps, when combined with soil bulk density data, Soil Fertility Index maps (Schaetzl et al. 2012) and/or nutrient gap (deficiency) maps. Such maps can be beneficial for non-specialist audiences who are nevertheless interested in spatial distributions of soil nutrients. The maps from this research could also be used as prior estimates that could be updated with more intensive local level sampling.

In addition to deriving higher-level products from this data set, combining these soil nutrient data with other continent-wide data sets will also yield insights. For example, data sets on weather (multiple years), farm management and root depth soil water (Leenaars et al. 2015) combined with data sets on crop distribution and yield, both actual and potential, will lead to insights about edaphic and agronomic drivers of yields gaps and associated nutrient gaps, or help policy makers target areas likely to undergo future nutrient depletion through crop removal and prevent areas that would otherwise fall below some critical nutrient level in the near to medium future. Other socio-economic data sets, such as health or income surveys, could be paired with these data to demonstrate how soil nutrient depletion can affect livelihoods and health outcomes, as well as to model the effects of predicted soil nutrient changes. Finally, this dataset could be combined with ecological data, such as biophysical inventories or NDVI data sets to refine our understanding of the role soil nutrients play in the heterogeneous and seemingly stochastically shifting plant community regimes of the semi-arid tropics, the underlying dynamics of which are still poorly understood (Murphy and Bowman 2012).

As we have already noted, probably the most serious limitation of this project was the high spatial clustering of points, i.e. under-sampling in countries with security issues or poor road infrastructures (tropical jungles, wetlands and similar). Fitting models with (only) 60 sites could result in many parts of Africa containing only extrapolated areas as topsoil data are predominantly collected/available for Eastern Africa (Ethiopia, Kenya, Uganda, Rwanda, Burundi, Tanzania), with large areas of relatively fertile soils developed from materials of volcanic origin located at relatively high altitude. More sampling points are certainly needed to improve spatial prediction models (and also to make the cross-validation more reliable), especially in West African soils developed in basement complexes (granites, gneisses, schists) and deposits and which are generally very much lower in soil nutrient contents. Because of high spatial clustering of points, and consequent extrapolation problems, the maps presented in this work should be used with caution. In that context, for the purpose of pan-African mapping it would be important to further optimize spreading of the sampling locations especially to increase representation of the geological and particularly the pedological feature space. This would increase sampling costs, but it might be the most efficient way to improve accuracy and usability of maps for the whole continent.

Also soil subsoil could be somewhat better represented. As the majority (>90%) of measurements refer to topsoil, unfortunately, we cannot tell if these soil-depth relationships are also valid for subsoil i.e. beyond 50 cm of depth and including the soil C horizon in weathering substrate. So also collecting soil nutrient measurements for depths beyond 50 cm could lead to interesting discoveries, especially when it comes to mapping organic Carbon and Nitrogen, soil alkalinity and similar.

## Conclusions

Spatial predictions of main macro- and micro-nutrients have been produced for soils of Sub-Saharan Africa using an international compilation of soil samples from various projects. Our focus was mainly on producing spatial prediction of extractable concentrations of soil nutrients (thus relative nutrient content estimates based on Mehlich-3 and compatible methods). For phosphorus we also produced maps of the total P content and for carbon and nitrogen we produced maps of organic component of the two elements.

The results of cross-validation showed that, apart from S, P and B, which seemed to be more difficult to model spatially using the given framework, significant models can be produced for most targeted nutrients (R-square between 40–85%; Table 1). Produced maps of soil macro- and micro-nutrients (Figs. 5, 6) could potentially be used for delineating areas of nutrient deficiency/sufficiency relative to nutrient requirements and as an input to crop modeling. Results of cluster analysis indicate that whole of SSA could be represented with ca. 20 classes (Fig. 10), which could potentially serve as the (objectively delineated) nutrient management zones.

The finally produced predictions represent a long-term (average) status of soil nutrients for a period from 1960–2016. The training data set could have been subset to more recently collected soil samples (2008–2016) to try to produce baseline estimates of soil nutrients for e.g. 2010. We have decided to use all available nutrient data instead, mainly to avoid huge sampling gaps, but also because our covariates cover longer time spans.

A limiting factor for mapping nutrients using the existing point data in Africa is a high spatial clustering of sampling locations with many countries/land cover and land use groups completely unrepresented (based on the Shannon Entropy Index map in Fig. 10). Logical steps towards improving prediction accuracies include: further collection of input (training) point samples, especially in areas that are under-represented or where the models perform poorly, harmonization of observations, addition of more detailed covariates specific to Africa, and implementation of full spatiotemporal statistical modeling frameworks (i.e. matching more exactly in time domain nutrient concentrations, crop yields and weather conditions).

Overlaying soil nutrient data with crop yield trials data shows that soil nutrients are indeed important for agricultural development with especially Mn, Zn, Al, B and Na, being listed high as the most important variables for prediction of crop yield (Fig. 11). If both nutrient maps and climatic images of the area are available, crop yields can be predicted with an average error of ±2:4 t ha^−1^ . If a more up-to-date field trial database was available, the model from Eq. (5) could have been used to produce more actual maps of potential yield (as compared to Fig. 12). Because the model from Eq. (5) can be used to produce almost infinite combinations of predictions, it would be also fairly interesting to serve the model as a web-service, i.e. so that users can inspect potential yields on demand (for arbitrary chosen combination of crop type, variant and application).

The gridded maps produced in this work are available under the Open Data Base licenses and can be downloaded from http://data.isric.org. These maps will be gradually incorporated into Web-services for soil nutrient data, so that also users on the field can access the data in real-time (i.e. through mobile phone apps and cloud services).

## Notes

1 http://data.worldbank.org/indicator/AG.LND.ARBL.ZS?locations=ZG.

2 https://github.com/ISRICWorldSoil/AfricaSoilNutrients.
